# Supplementary material for: Nasogastric Tube Feeding in Anorexia Nervosa: A Propensity Score-Matched Analysis on Clinical Efficacy and Treatment Satisfaction
Source: Nutrients. 2024 May 29;16(11):1664. doi: 10.3390/nu16111664 (PMC11174568; doi:10.3390/nu16111664)
Supplement: Supplementary file 1 [file nutrients-16-01664-s001.zip › nutrients-3006195-supplementary.pdf]

Nasogastric Tube Feeding in Anorexia Nervosa: A Propensity Score-Matched Analysis on Clinical Efficacy and Treatment Satisfaction

Matteo Martini, Paola Longo, Clara Di Benedetto, Matteo Panero, Nadia Delsedime, Giovanni Abbate-Daga * and Federica Toppino

Eating Disorders Center, Department of Neuroscience "Rita Levi Montalcini", University of Turin, Via Cherasco 11, 10126 Turin, Italy

* Correspondence: giovanni.abbatedaga@unito.it

SUPPLEMENTARY MATERIALS

Table S1. Comparison of end of treatment non-completers in the two groups

**Characteristic**	**NGT**, N = 27[1]	**non-NGT**, N = 25[1]	**p-value**[2]
number of hospitalization			0.9
1	21 (78%)	19 (76%)	
2	6 (22%)	6 (24%)	
Diagnosis			0.6
AN-BP	8 (30%)	9 (36%)	
AN-R	19 (70%)	16 (64%)	
BMI	13.94 (1.99)	13.81 (1.97)	0.8
caloric intake	593 (429)	614 (379)	0.8
duration of illness (years)	8 (10)	5 (6)	0.2
psychiatric comorbidity	10 (37%)	7 (28%)	0.5
personality disorder	6 (22%)	4 (16%)	0.7
length of stay (days)	40 (29)	42 (23)	0.8
EDE-Q restraint	3.93 (1.96)	3.33 (2.36)	0.3
EDE-Q eating concern	3.59 (1.25)	2.76 (1.79)	0.062
EDE-Q shape concern	4.85 (1.24)	4.19 (1.79)	0.13
EDE-Q weight concern	4.54 (1.44)	3.55 (1.91)	0.042
EDE-Q global score	4.23 (1.33)	3.46 (1.80)	0.089
caloric intake - discharge	1,367 (389)	1,379 (301)	>0.9

**Characteristic**	**NGT**, N = 27[1]	**non-NGT**, N = 25[1]	**p-value**[2]
BMI increase	0.68 (0.56)	0.59 (0.73)	0.6

[1]n (%); Mean (SD)

[2]Pearson's Chi-squared test; Welch Two Sample t-test; Fisher's exact test

Abbreviations: AN-R=Anorexia Nervosa - Restricting Type; AN-BP=Anorexia Nervosa Binge-Purging Type; BMI=Body Mass Index; EDE-Q=Eating Disorder Examination Questionnaire; NGT=Nasogastric Tube

Figure S1. Directed Acyclic Graphs (DAGs): variables in white represent the minimal set of adjustment required to estimate the effect of the exposure (enteral therapy) on the outcomes

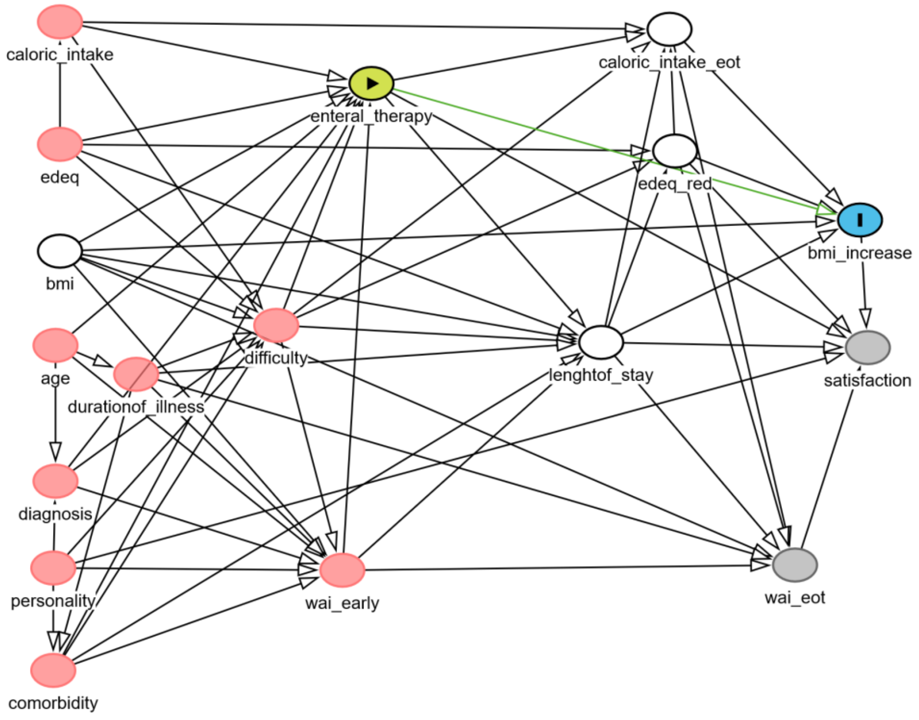

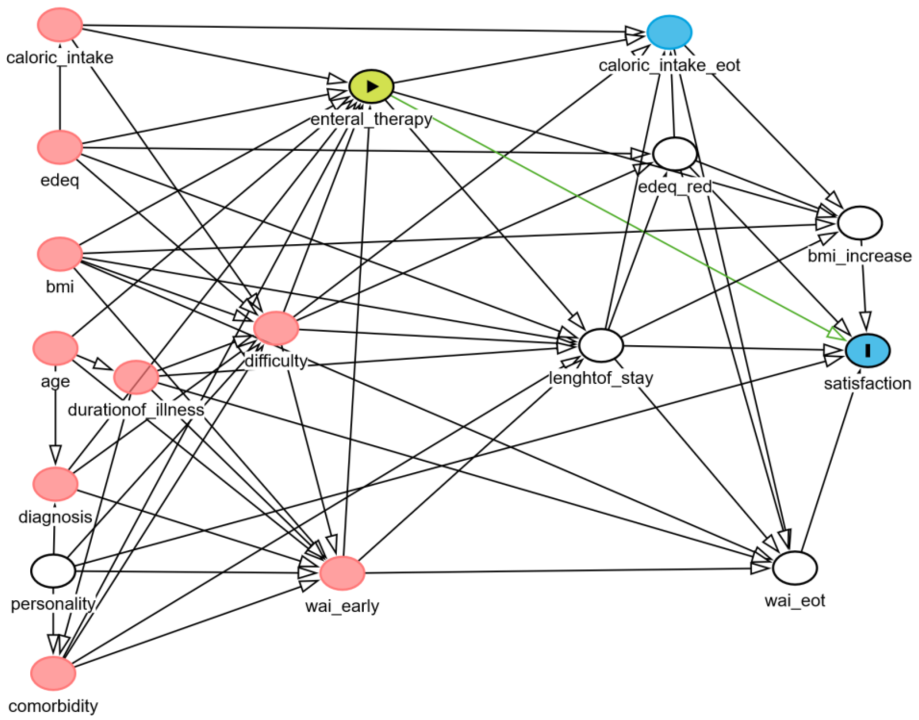

Legend: The exposure is depicted as a green circle with a black triangle in the middle. The outcome is represented by a blue circle with a black rectangle in the middle. Red circles are ancestor of both exposure and outcome, whereas blue circles are ancestor of outcome. White circles are the adjusted variable, and grey other variables not influencing the causal path of interest. The green arrow identifies the causal path. Biasing paths would appear as purple arrows. In this case, multiple biasing paths would be evident if adjusted variables would not be defined as such (i.e., not conditioned on in the analysis).

Abbreviations: BMI=Body Mass Index; EDE-Q=Eating Disorder Examination Questionnaire; WAI=Working Alliance Inventory; EOT=End Of Treatment

Table S2. Sample sizes

	Control	Treated
All (ESS)	243	97
All	243	97
Matched (ESS)	97	97
Matched	97	97
Unmatched	146	0
Discarded	0	0

Table S3. Summary of balance for matched data

	Means Treated	Means Control	Std. Mean Diff.	Var. Ratio	eCDF Mean	eCDF Max	Std. Pair Dist.
Distance	0.35	0.34	0.08	1.27	0.01	0.09	0.09
Age	24.17	24.57	-0.05	0.89	0.05	0.12	1.03
Occurrence	1.38	1.38	0.00	0.80	0.03	0.04	0.60
Duration of illness	6.69	7.06	-0.04	1.20	0.03	0.16	0.99
Diagnosis (AN-BP)	0.66	0.62	0.09	NA	0.04	0.04	1.22
Diagnosis (AN-R)	0.34	0.38	-0.09	NA	0.04	0.04	1.22
Psychiatric Comorbidity	0.44	0.46	-0.04	NA	0.02	0.02	0.95
Personality Disorder	0.23	0.19	0.10	NA	0.04	0.04	0.79
BMI	13.99	14.01	-0.01	0.87	0.03	0.10	1.22
EDE-Q global score	3.98	4.00	-0.01	1.17	0.02	0.08	0.84
Caloric intake	600.41	632.68	-0.09	1.27	0.03	0.14	0.72

Abbreviations: AN-R=Anorexia Nervosa - Restricting Type; AN-BP=Anorexia Nervosa Binge-Purging Type; BMI=Body Mass Index; EDE-Q=Eating Disorder Examination Questionnaire

Table S4. Balance success.

	Type	Diff.Adj	M.Threshold
Distance	Distance	0.083010726	Balanced, <0.1
Age	Contin.	-0.047338510	Balanced, <0.1
Occurrence	Contin.	0.000000000	Balanced, <0.1
Duration of illness	Contin.	-0.041464799	Balanced, <0.1
Diagnosis subtype	Binary	-0.041237113	Balanced, <0.1
Psychiatric Comorbidity	Binary	-0.020618557	Balanced, <0.1
Personality Disorder	Binary	0.041237113	Balanced, <0.1
BMI	Contin.	-0.009898188	Balanced, <0.1
EDE-Q global score	Contin.	-0.011685336	Balanced, <0.1
Caloric intake	Contin.	-0.085422129	Balanced, <0.1

Figure S2. Distribution of propensity scores

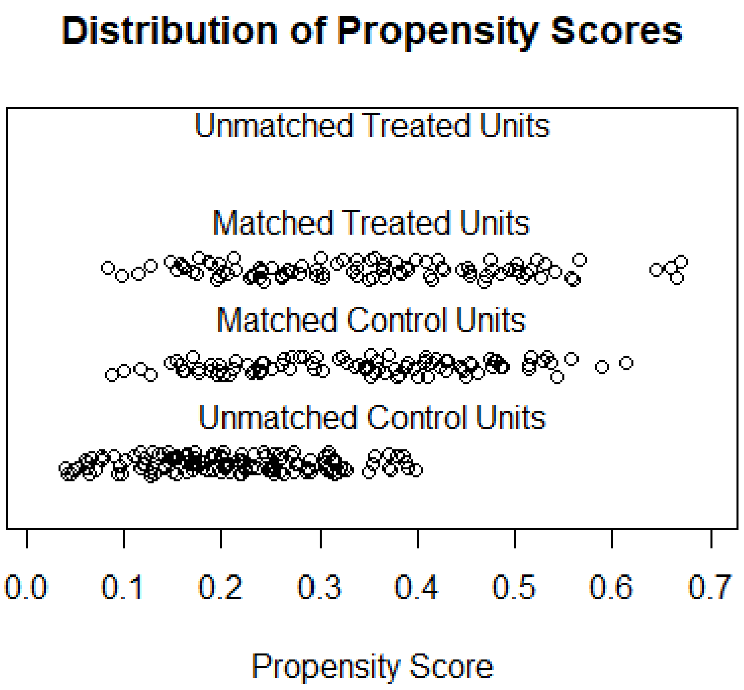
Distribution of Propensity Scores
Unmatched Treated Units
Matched Treated Units
Matched Control Units
Unmatched Control Units
0.0
0.1
0.2
0.3
0.4
0.5
0.6
0.7
Propensity Score

Figure S3. Model check for BMI increase

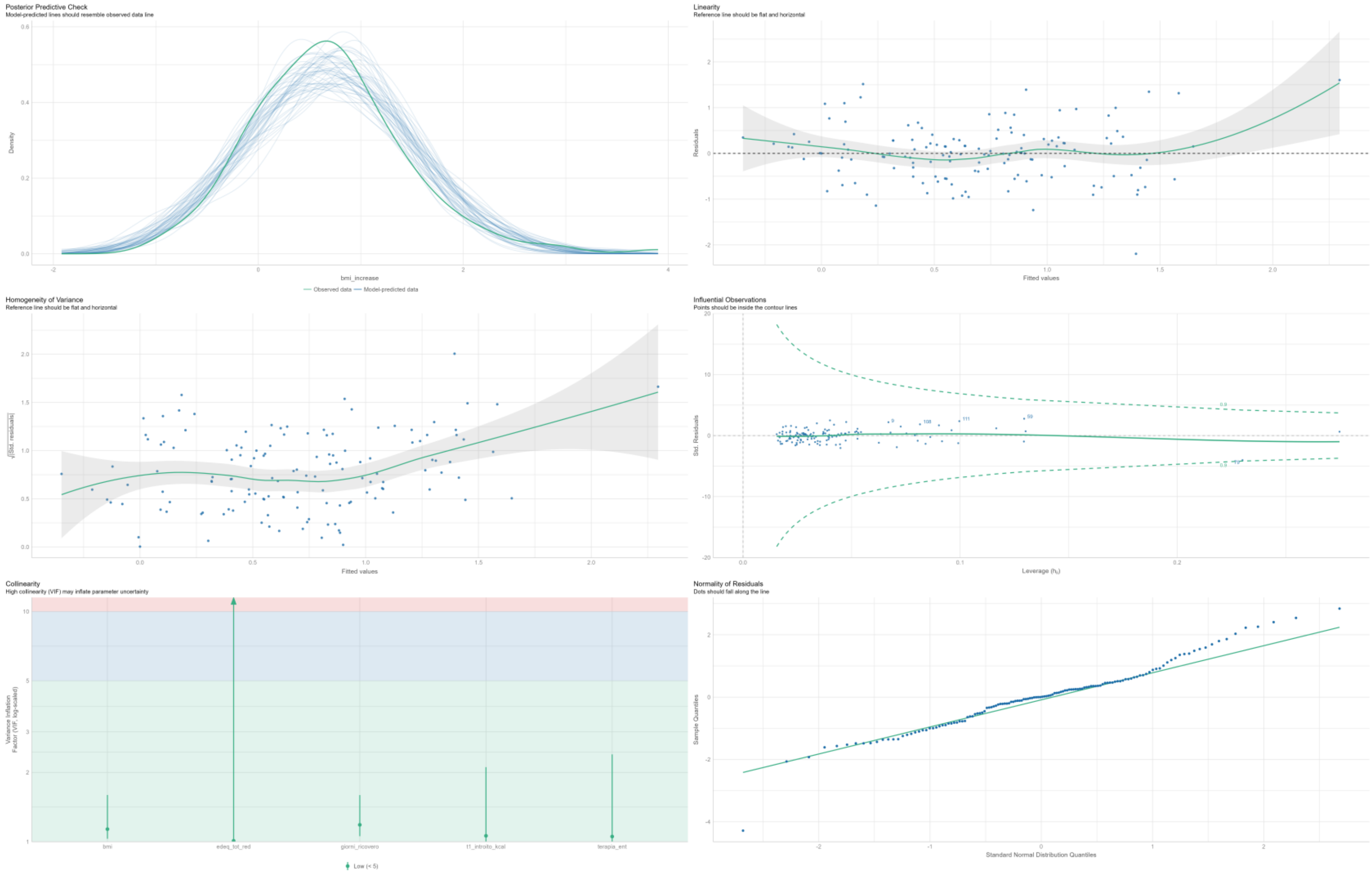

Figure S4. Model check for End Of Treatment usefulness

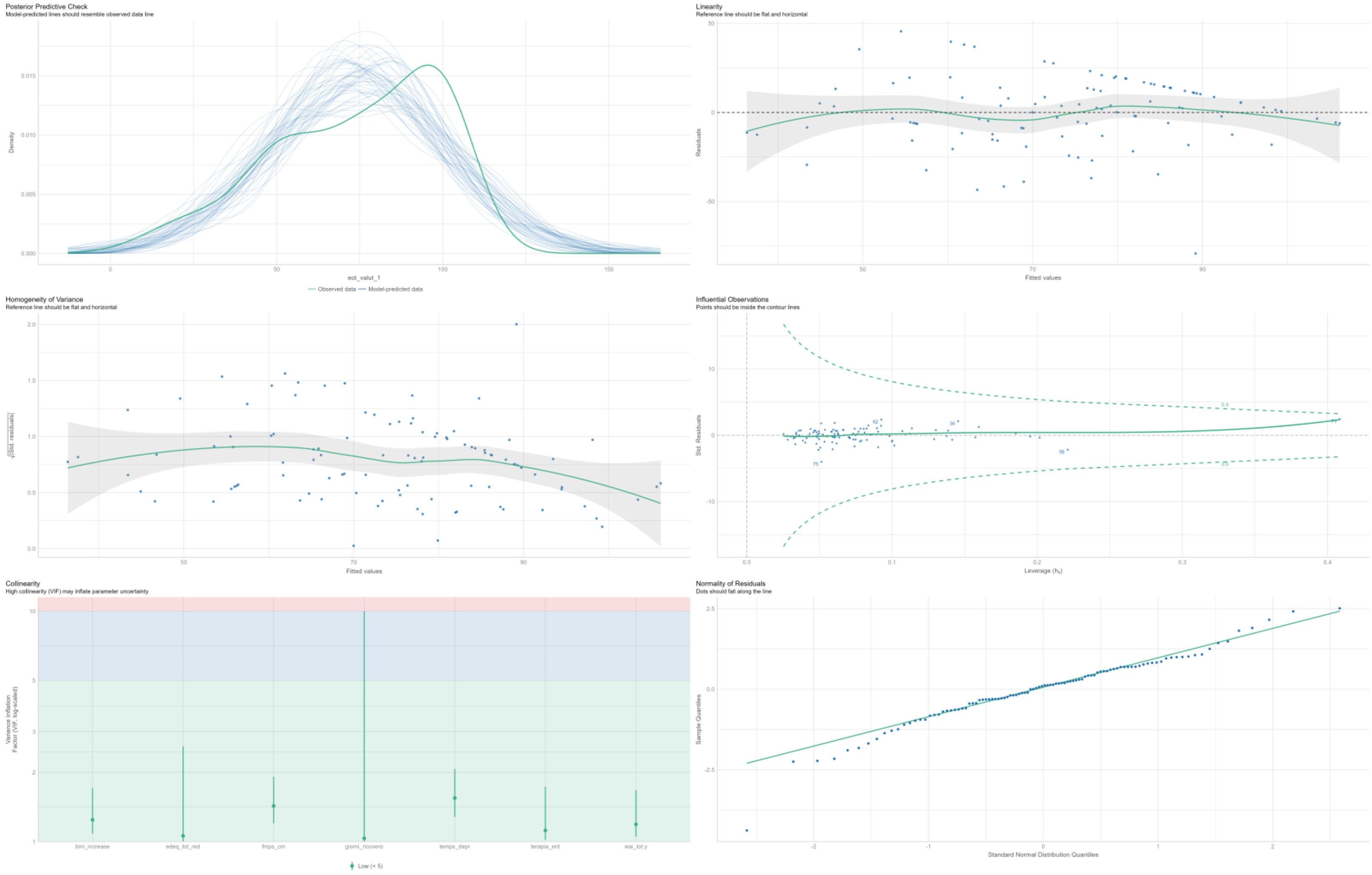

Abbreviations: eot_valut_1=end of treatment usefulness

Figure S5. Model check for End Of Treatment satisfaction

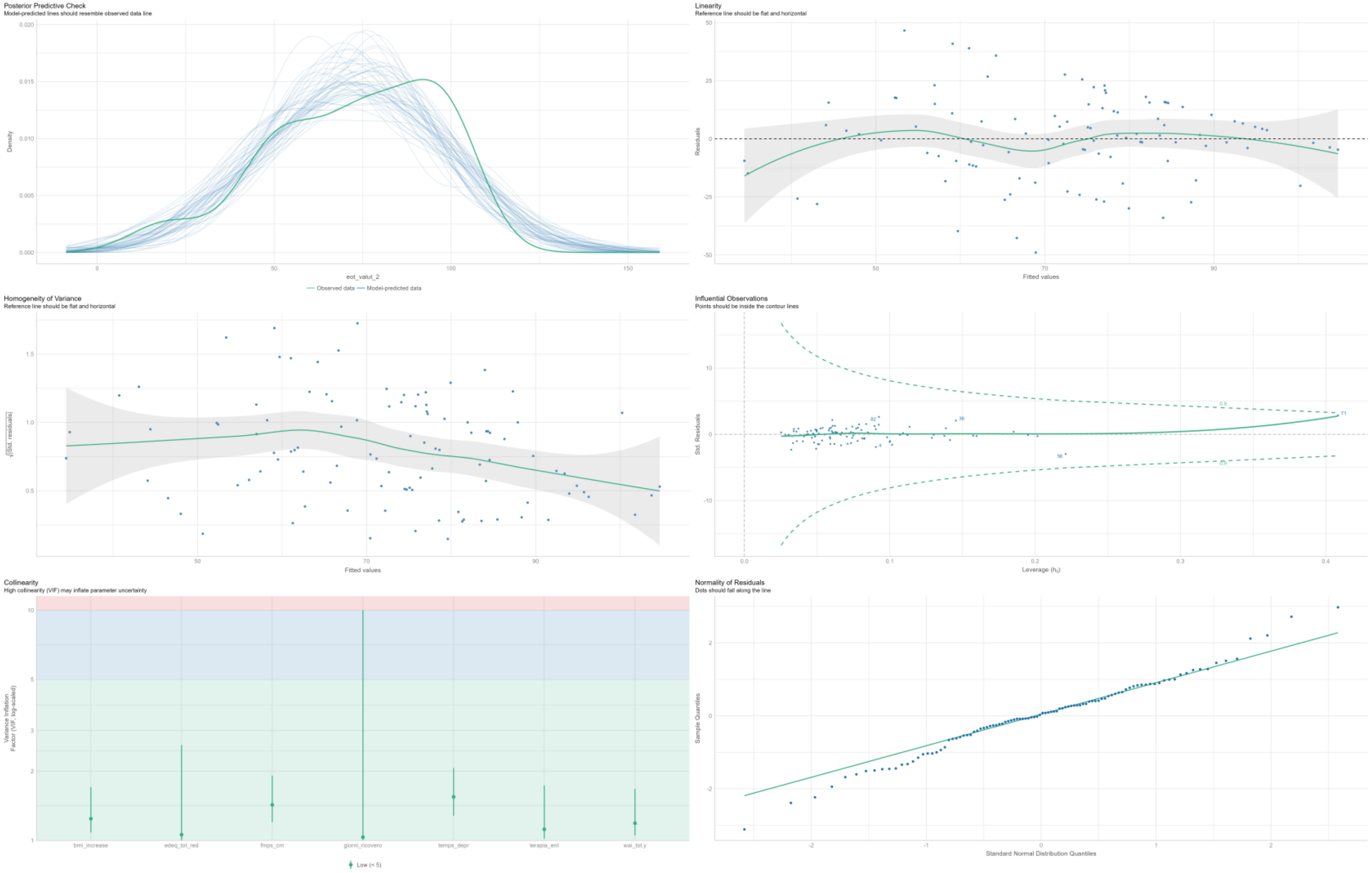

Abbreviations: eot_valut_2=end of treatment satisfaction

Figure S6. Model check for End Of Treatment subjective improvement

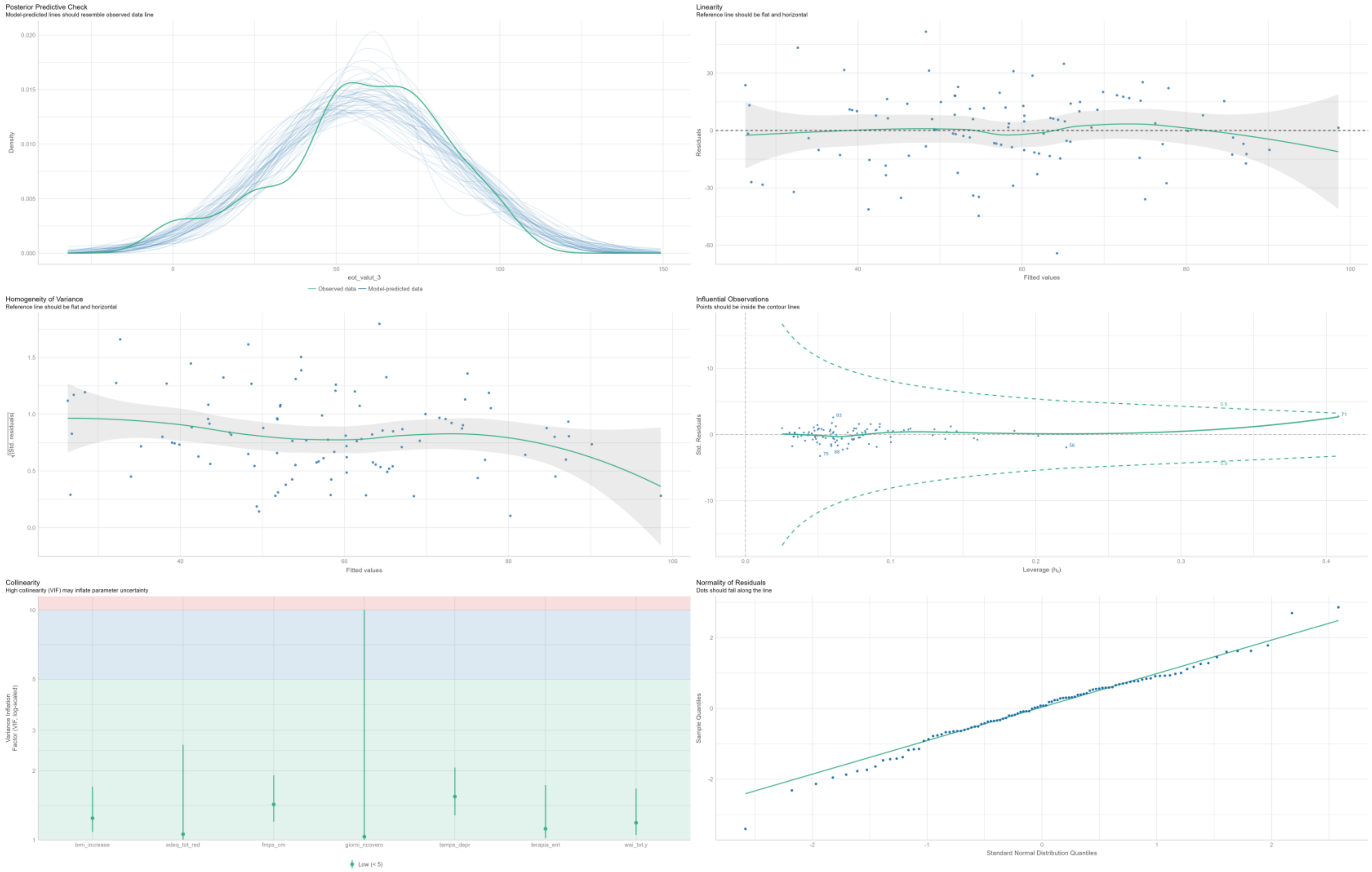

Abbreviations: eot_valut_3=end of treatment subjective improvement

Table S5. Personality traits comparison

	Total Sample			Matched		
**Characteristic**	**NGT**, N = 64[1]	**non-NGT**, N = 171[1]	**p-value**[2]	**NGT**, N = 65[1]	**non-NGT**, N = 69[1]	**p-value**[2]
**FMPS concern over mistakes**	32 (10)	30 (10)	0.2	32 (10)	33 (9)	0.6
FMPS personal standards	26 (6)	25 (6)	0.14	26 (6)	25 (6)	0.3
FMPS parental expectations	11 (4)	11 (5)	0.8	11 (5)	11 (6)	0.9
FMPS parental criticism	10 (4)	10 (4)	0.4	10 (4)	10 (4)	>0.9
FMPS doubts about actions	13 (4)	13 (4)	0.5	13 (4)	14 (4)	0.3
FMPS organization	25 (5)	24 (5)	0.4	24 (5)	24 (5)	0.6
**TEMPS-A depressive temperament**	14 (4)	13 (4)	0.2	13 (4)	14 (4)	0.2
TEMPS-A cyclothymic temperament	10 (5)	9 (5)	0.2	10 (5)	10 (5)	0.8
TEMPS-A hyperthymic temperament	7 (4)	7 (4)	0.7	7 (4)	6 (4)	0.025
TEMPS_A irritable temperament	7 (4)	6 (4)	0.3	7 (4)	7 (4)	0.9
TEMPS-A anxious temperament	14 (5)	13 (6)	0.2	15 (5)	15 (6)	0.8

[1]Mean (SD)

[2]Welch Two Sample t-test

Abbreviations: BMI=Body Mass Index; EDE-Q=Eating Disorder Examination Questionnaire; FMPS=Frost Multidimensional Perfectionism Scale; TEMPS-A=Temperament Evaluation of Memphis, Pisa, Paris, and San Diego Autoquestionnaire; NGT=Nasogastric Tube
